# Recent Advances and Contradictions in the Study of the Individual Roles of Ubiquitin Ligases That Regulate RIG-I-Like Receptor-Mediated Antiviral Innate Immune Responses

**DOI:** 10.3389/fimmu.2020.01296

**Published:** 2020-06-24

**Authors:** Hiroyuki Oshiumi

**Affiliations:** Department of Immunology, Faculty of Life Sciences, Graduate School of Medical Sciences, Kumamoto University, Kumamoto, Japan

**Keywords:** RIG-I, MDA5, innate immunity, ubiquitin, virus, type I interferon

## Abstract

RIG-I and MDA5 are cytoplasmic viral RNA sensors and are essential for antiviral innate immune responses, such as type I interferon production. Post-translational modification is critical for the activation and inactivation of RIG-I and MDA5. At least seven ubiquitin ligases have been reported to be involved in either K63- or K48-linked polyubiquitination of RIG-I and MDA5, and these ubiquitin ligases are further regulated by other factors. TRIM25 is an E3 ubiquitin ligase that delivers a K63-linked polyubiquitin moiety to the caspase activation and recruitment domains (CARDs) of RIG-I, thereby activating the antiviral innate immune response. Recent studies have shown that NDR2, ZCCHC3, and Lnczc3h7a promote TRIM25-mediated RIG-I activation. Riplet is another ubiquitin ligase that mediates the K63-linked polyubiquitination of the C-terminal domain (CTD) of RIG-I; however, it was also reported that Riplet delivers the K63-linked polyubiquitin moiety to the CARDs of RIG-I as well as to the CTD, thereby activating RIG-I. Further, there are several factors that attenuate the activation of RIG-I and MDA5. RNF125, TRIM40, and c-Cbl mediate K48-linked polyubiquitination and induce degradation of RIG-I and/or MDA5. USP21 and CYLD remove the K63-linked polyubiquitin chain from RIG-I, and NLRP12 inhibits polyubiquitin-mediated RIG-I activation. Although these new regulators have been reported, their distinctive roles and functional differences remain elusive, and in some cases, studies on the topic are contradictory to each other. In the present review, recent studies related to post-translational modifications of RIG-I and MDA5 are summarized, and several controversies and unanswered questions in this field are discussed.

## Introduction

Cytoplasmic viral double-stranded RNA (dsRNA) is recognized by RIG-I-like receptors (RLRs), which include RIG-I, MDA5, and LGP2 (1, 2). 5′ tri- and di-phosphate of dsRNA are crucial for the recognition of viral RNA by RIG-I, whereas 5′phosphate of dsRNA is dispensable for MDA5 activation (3–5). The expression of RLRs is induced after viral infection in various types of cells. Dendritic cells and macrophages as well as epithelial cells utilize RIG-I and MDA5 to sense viral RNA and to produce type I interferon (IFN) at an early phase of viral infection (6, 7). An accumulating body of evidence has shown that RLRs play a crucial role in antiviral innate immune responses (5, 8).

After recognition of viral dsRNA, RIG-I and MDA5 trigger the signals that induce the expression of type I IFN and other pro-inflammatory cytokines via a MAVS adaptor molecule, which is also called IPS-1, CARDIF, and VISA (9–12). While RIG-I recognizes relatively short dsRNA molecule (<1 kbp), MDA5 recognizes longer dsRNAs (>1 kbp) (13); thus, these proteins exhibit different specificities to viral infections (7). For instance, influenza A virus, hepatitis C virus, and respiratory syncytial virus are preferentially recognized by RIG-I, whereas encephalomyocarditis virus and Mengovirus are recognized by MDA5 (14).

The RIG-I and MDA5 proteins carry two caspase activation and recruitment domains (CARDs) at their N-terminal regions, which are essential for MAVS activation (8). In contrast, LGP2 does not possess any CARDs and plays only a regulatory role in the activation of RIG-I and MDA5 (8). The C-terminal domains (CTDs) of RIG-I and MDA5 recognize viral RNA, and the proteins assemble along dsRNA to make a nucleoprotein filament (15, 16). The formation of the nucleoprotein filament leads to inter-molecular association of RIG-I CARDs, which results in the formation of a 2CARD tetramer structure (17). This 2CARD tetramer structure functions as a core of MAVS oligomerization, resulting in MAVS prion-like fiber formation (18). The MAVS fibers on mitochondria are essential for activating downstream protein kinases, such as TBK1 and IKK-ε (18). Activated protein kinases phosphorylate an IRF3 transcription factor to induce type I IFN expression (19). Recently, we identified a scaffold protein, zyxin, that stabilizes the interaction between MAVS, RIG-I, and MDA5 (20). Considering that the zyxin protein localizes to focal adhesions and regulates actin filament assembly (21, 22), it might function as the scaffold required to start the assembly and filament formation of MAVS as well as actin.

There are many regulators of RIG-I and MDA5, such as ubiquitin ligases and protein kinases. Further, those ubiquitin ligases are regulated by several host and viral proteins. In the present review, we summarize recent findings related to the ubiquitin-mediated regulation of RLRs.

## Ubiquitin Ligases That Mediate K63-Linked Polyubiquitination OF RIG-I

**Figure 1:**
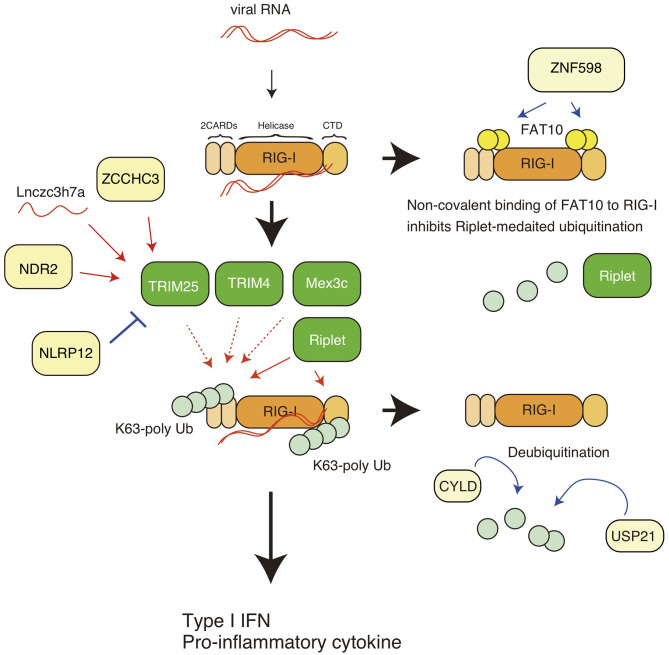
K63-linked polyubiquitin chain-mediated regulation of RIG-I. The RIG-I protein comprises the N-terminal caspase activation and recruitment domains (CARDs), an RNA helicase domain, and the C-terminal domain (CTD). K63-linked polyubiquitination leads to activation of RIG-I, leading to the expression of type I IFN and other pro-inflammatory cytokines. TRIM25, Riplet, Mex3c, and TRIM4 are reported to mediate K63-linked polyubiquitination of the CARDs, and Riplet mediates the polyubiquitination of both the CARDs and the CTD. ZNF598 promotes the binding of FAT10 to RIG-I, thereby inhibiting Riplet-mediated ubiquitination of RIG-I. CYLD and USP remove the polyubiquitin chain from RIG-I. The ZCCHC3 and NDR2 protein promote TRIM25-medaited polyubiquitination of RIG-I. A non-coding long RNA, Lnczc3h7a, promotes TRIM25-medated RIG-I activation.

The RIG-I protein harbors several post-translational modifications, and polyubiquitin modification is crucial for RIG-I activation and degradation. Gack et al. first reported that RIG-I harbors K63-linked polyubiquitination, leading to the activation of downstream signaling (23) (Figure 1). The K172 residue of the RIG-I CARDs is ubiquitinated, which is essential for the induction of type I IFN expression (23). Later studies showed that both covalent binding and non-covalent binding of the K63-linked polyubiquitin chain to the K172 residue are sufficient to induce the RIG-I activation (17, 24). The K63-linked polyubiquitin chains stabilize the RIG-I 2CARD tetramer (17). TRIM25 was first reported to be responsible for delivering the polyubiquitin moiety to RIG-I CARDs (23, 24).

Riplet (also called RNF135 and Reul) is another ubiquitin ligase (25). Since knockout (KO) of Riplet results in severe defects in the *in vivo* antiviral innate immune response, such as impaired type I IFN production and decreased survival of virally infected mice (26), Riplet-mediated RIG-I ubiquitination is crucial for *in vivo* antiviral innate immune responses. The Riplet protein preferentially binds to the RIG-I CTD and mediates K63-linked polyubiquitination at K788, K849, K851, K888, K907, and K909 (27, 28). It has been shown that Riplet promotes the binding of TRIM25 to RIG-I, thereby augmenting TRIM25-mediated RIG-I activation (28). Thus, a sequential ubiquitination model has been postulated (25, 28). However, recent studies have reported that Riplet ubiquitinated multiple sites of RIG-I, including the 2CARDs, and produced unanchored polyubiquitin chains, thereby promoting RIG-I activation (29, 30).

Many studies have reported that TRIM25 is responsible for RIG-I ubiquitination and activation, whereas several recent studies have shown that Riplet, but not TRIM25 is essential for RIG-I activation (30, 31). In addition, there are several reports illustrating the RIG-I-independent antiviral activities of TRIM25 (32, 33). On the other hand, it has been reported that Riplet can activates IRF3 transcription factor in a RIG-I-independent manner (34). Since there are differences in the experimental conditions between recent studies, and other possible explanations which have been recently discussed in more detail (35), the apparent contradictions should be reconciled by further studies. Despite these recent contradictory results, many studies have identified regulatory factors (proteins and lncRNAs) that regulate TRIM25-medaited RIG-I K63-linked polyubiquitination and activation, and over the past several years, many viral antagonists of TRIM25 have been discovered which function to inhibit the K63-linked polyubiquitination and signaling of RIG-I (as described below). In addition to TRIM25 and Riplet, Mex3c and TRIM4 ubiquitin ligases have been reported to be involved in the K63-linked polyubiquitination and activation of RIG-I (36, 37). Amino acid substitution at K45, K99, and K169 attenuated Mex3c-mediated polyubiquitination of RIG-I (36), and TRIM4 targeted K164 and K172 of RIG-I (37) (Figure 1). However, Shi et al. reported that knockout of Mex3c and TRIM4 did not affect the activation of MAVS upon stimulation (38). These apparent discrepancies should be addressed in further studies to determine why RIG-I requires four ubiquitin ligases and why previous studies apparently contradict to each other.

## Ubiquitin Ligases That Mediate K48-Linked Polyubiquitination OF RIG-I

**Figure 2:**
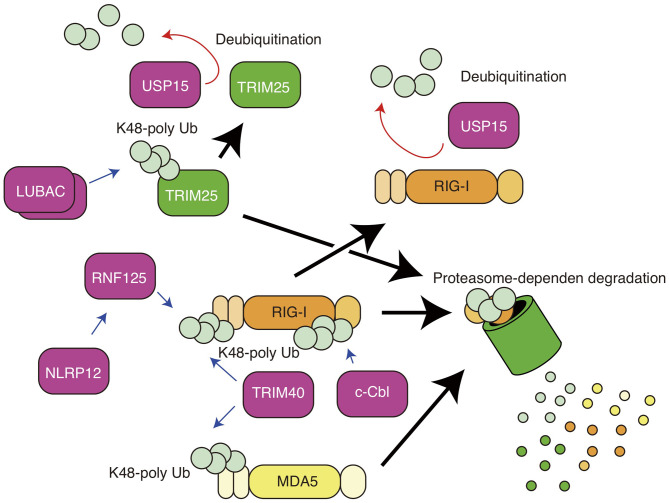
K48-linked polyubiquitin chain-mediated regulation of RIG-I and MDA5. K48-linked polyubiquitination of RIG-I and TRIM25 leads to proteasomal degradation of the proteins. RNF125 mediates K48-linked polyubiquitination of RIG-I, and NLRP12 promotes TRIM25-mediated ubiquitination. TRIM40 conjugates K48-linked polyubiquitin chain to both RIG-I and MDA5. c-Cbl mediates K48-linked polyubiquitination of RIG-I. USP15 removes RNF125-mediated polyubiquitin chain.

The RIG-I protein is also regulated by K48-linked polyubiquitination, which is well-known to lead to protein degradation in a proteasome-dependent manner (Figure 2). RNF125 is a ubiquitin ligase that mediates the K48-linked polyubiquitination of RIG-I, thereby promoting the degradation of the protein via proteasomes (39). RNF125 attenuates RIG-I-induced IFN-β production (39). c-Cbl is an E3 ubiquitin ligase and is recruited to the cytoplasmic tail of Siglec-G together with SHP2 (40). c-Cbl mediates K48-lined polyubiquitination of RIG-I at the K813 residue and thereby promotes RIG-I degradation (40). TRIM40 is a member of the TRIM family and binds to the CARDs of RIG-I (41). TRIM40 promotes K27- and K48-linked polyubiquitination of RIG-I (41). Although the *in vivo* roles of RNF125 and c-Cbl in the RIG-I-mediated innate immune response remain unclear, it has been shown that knockout of TRIM40 enhances RIG-I-mediated type I IFN production and improves the survival of mice infected with vesicular stomatitis virus, which is recognized by RIG-I (41). Since excessive activation of RIG-I leads to autoimmune disorders (42), these ubiquitin ligases are expected to be important in preventing excessive RIG-I activation.

## Regulators of Ubiquitin Ligase Targeting RIG-I

FAT10 is a ubiquitin-like modifier and is also involved in the control of RIG-I activation (43, 44). In general, covalent binding of FAT10 to a target protein leads to degradation of the target protein via proteasome (45, 46). A previous study reported that non-covalent binding of FAT10 to RIG-I decreased the solubility of the RIG-I protein, resulting in attenuation of RIG-I-mediated cytokine expression (44).

The ZNF598 protein is known to exhibit E3 ubiquitin ligase activity and to be required for the ubiquitination of ribosome components for ribosome quality control (47, 48). DiGiuseppe et al. first reported that ZNF598 decreases the expression of antiviral genes, such as PKR, ISG56, and MxA (49). Recently, we showed that ZNF598 promoted non-covalent binding of FAT10 to RIG-I, thereby inhibiting Riplet-mediated K63-linked polyubiquitination of RIG-I (50).

There are several other mechanisms that inhibit K63-linked polyubiquitin chain-mediated RIG-I activation. USP21 is a deubiquitinating enzyme that removes the K63-linked polyubiquitin chain from RIG-I, resulting in the attenuation of innate immune responses (51). CYLD is another deubiquitinating enzyme that also removes the K63-linked polyubiquitin chain from RIG-I, and thus functions as a negative regulatory factor (52). Those activities might be promoted by FAT10, because the binding of FAT10 to RIG-I leads to reduced polyubiquitination of RIG-I (50).

Previous studies have reported several factors that regulate the activities of RIG-I via TRIM25. TRIM25 ubiquitin ligase itself is also regulated by ubiquitination. Liner ubiquitin assembly complex (LUBAC), comprising the HOIL-1L and HOIP proteins, conjugates K48-linked polyubiquitin chains to TRIM25, resulting in proteasome-dependent degradation of TRIM25 (53). USP15 is a ubiquitin-specific protease and cleaves the K48-linked polyubiquitin chain of TRIM25 (54).

NLRP12, a member of the Nod-like receptor (NLR) family, is expressed in dendritic cells and neutrophils (55) and recognizes *Yersinia pestis* infection, leading to inflammasome-mediated IL-1β processing and the production of mature IL-1β (56). NLRP12 also has inflammasome-independent functions that regulates RIG-I. The nucleotide-binding domain of NLRP12 physically interacts with TRIM25 and attenuates TRIM25-mediated K63-linked polyubiquitination (57). In addition, NLRP12 enhances the RNF125-mediated K48-linked polyubiquitination of RIG-I; thus, NLRP12 KO augments serum type I IFN levels and reduces viral titers in brain after intranasal inoculation with vesicular stomatitis virus (57).

NDR2 is a protein kinase and is involved in a variety of biological processes, such as apoptosis, tumorigenesis, and cell division (58). NDR2 deficiency decreases RIG-I-mediated type I IFN production, resulting in increased susceptibility to vesicular stomatitis virus and influenza A virus infection (59). The NDR2 protein associate with RIG-I and TRIM25 and promotes the TRIM25-mediated K63-linked polyubiquitination of RIG-I (59). ZCCHC3 is a zinc-finger protein and functions as a co-sensor of cGAS, which is a cytosolic DNA sensor. The protein binds to cytoplasmic viral RNA and acts as a co-receptor of RIG-I. Biochemical studies have shown that ZCCHC3 recruits TRIM25 to RIG-I, thereby facilitating K63-linked polyubiquitination (60). In addition, long non-coding RNA (lncRNA) also regulates RIG-I activation via TRIM25. For example, the lncRNA Lnczc3h7a binds TRIM25 (61). The association between lncRNA and TRIM25 occurs even in resting cells and promotes TRIM25-mediated K63-linked polyubiquitination of RIG-I during viral infection (61). The roles of those factors are summarized in Figures 1, 2. Since there are contradictory reports related to TRIM25 function (30, 31), further studies of those RIG-I regulators, such as FAT10, NDR2, ZCCHC3, and NLRP12, might reconcile the apparent contradictions.

## Post-Translational Modification OF MDA5

The MDA5 protein is regulated by phosphorylation (62). In resting cells, the 2CARDs and the CTD of MDA5 are phosphorylated to suppress abnormal activation (63). After recognition of a viral RNA, phosphatase 1 (PP1) dephosphorylates MDA5, resulting in the activation of its downstream signaling (63). Since excessive activation of MDA5 causes systemic lupus erythematosus-like autoimmune diseases (64), its phosphorylation is thought to be important for the prevention of harmful MDA5 activation in non-infected cells. RIOK3, a protein kinase, phosphorylates the CTD of MDA5 and enhanced MDA5-mediated cytokine expression (65). However, the CARDs of MDA5 are not phosphorylated by RIOK3, and thus it is expected that there is another protein kinase that phosphorylates the CARDs of MDA5.

The MDA5 protein is also polyubiquitinated. As in RIG-I protein, the CARDs of MDA5 bind to K63-linked polyubiquitin chains. The K174 residue of the MDA5 CARDs is essential for this binding; a ubiquitination-defective K174A mutant of MDA5 failed to induce type I IFN expression (66). However, which ubiquitin ligase is responsible for K63-polyubiquitination remains unclear. Zhao et al. reported that TRIM40 binds to the CARDs of MDA5 and promotes K27- and K48-linked polyubiquitination of MDA5, leading to proteasomal degradation of the protein (41). Further studies are required to fully reveal the role of ubiquitin in the MDA5 activation.

## Viral Proteins Targeting Ubiquitin Ligases

Several viral proteins target ubiquitin ligases to escape RIG-I-mediated innate immune responses. The NS1 protein of influenza A virus attenuates the IRF3 activation that is required for type I IFN expression (67). Gack et al. have shown that NS1 binds to TRIM25 and Riplet and attenuates the K63-linked polyubiquitination of RIG-I (68, 69). Hepatitis C virus persistently infects the host liver for several decades and can evade innate immune responses. Viral NS3-4A proteases cleaves viral poly-protein to produce mature viral proteins, and it also cleaves the MAVS adaptor, resulting in the release of MAVS from the mitochondrial outer membrane and thus inhibition of RIG-I-mediated type I IFN expression (10). The hepatitis C virus NS3-4A protein also binds to Riplet and inhibits Riplet-dependent antiviral activities (28, 34). Although NS3-4A cleaves and degrade Riplet in a protease activity-dependent manner, it has bee also postulated that there is a RIG-I-independent Riplet antiviral activity, attenuated by NS3-4A, plays a role (28, 34). RIG-I is able to recognize DNA virus infection (70, 71), and it has been shown that RIG-I senses HSV-1 infection via RNA5SP141 (72). Human papilloma virus E6 oncoprotein targets USP15 and TRIM25, thereby inhibiting RIG-I-mediated antiviral activities (73). In addition, it is also reported that viral proteins of EB virus antagonizes TRIM25 function (74). Considering that viruses have evolved to escape host innate immune responses (75), these observations support the notion that Riplet and TRIM25 are crucial for antiviral innate immune responses.

## Perspective

An accumulating body of evidence has shown that ubiquitination is a key post-translational modification for RIG-I (62). At least four ubiquitin ligases are reported to be required for K63-linked polyubiquitination of RIG-I. Among these ligases, the antiviral activities of Riplet and TRIM25 have been confirmed by several groups. Recent studies have reported that TRIM25 is dispensable for RIG-I ubiquitination (30, 31, 38); however, it is still possible that other ubiquitin ligases, such as Mex3c and TRIM4, play redundant roles and thus compensate for a lack of TRIM25. To test this possibility, it will be necessary to uncover the underlying molecular mechanism via analysis of double or triple KO of TRIM25, Mex3c, and TRIM4. It is also possible that Riplet and TRIM25 regulate RIG-I in a cell-type specific manner. Another possibility is that the expression levels of ZCCHC3, NDR2, and NLRP12 affect the dependency on TRIM25. Further studies are required to reconcile the apparent discrepancy. On the other hand, the question of why there are so many factors that regulate RIG-I is yet to be answered. ZNF598, RNF125, c-Cbl, and TRIM40 are negative regulators of RIG-I, but their distinct roles remain elusive. Their redundancy and functional differences should be further investigated to provide greater insight into the regulation of RIG-I.

## Author Contributions

The author confirms being the sole contributor of this work and has approved it for publication.

## Conflict of Interest

The author declares that the research was conducted in the absence of any commercial or financial relationships that could be construed as a potential conflict of interest.
